# Potential factors causing failure of whole plant nettle (*Urtica cannabina*) silages

**DOI:** 10.3389/fmicb.2022.1113050

**Published:** 2023-01-11

**Authors:** Rongzheng Huang, Yongcheng Chen, Chunhui Ma, Yuxin Chai, Shuan Jia, Fanfan Zhang

**Affiliations:** Grassland Science, School of Animal Technology, Shihezi University, Shihezi, China

**Keywords:** nettle, bacterial community, metabolites, silage, *Lactobacillus*

## Abstract

**Introduction:**

Nettle is kind of new feed resources and benefit for animal production. However, a few studies observed that quality of nettle silage was poor under naturally fermentation. Consider of microbial activity was the mainly factors for fermentation characteristics of silage.

**Methods:**

Thus, the present study investigated the potential factors causing nettle silage failure through metabolome and bacterial community composition analyses during ensiling.

**Results:**

During ensiling, the pH was >6.22, and water-soluble carbohydrate and organic acid contents stabilized after 7 d. At the genus level, *Enterococcus*, *Weissella*, and *Pediococcus* were the dominant bacteria (relative abundance were 30.06–39.39, 17.29–23.34, and 3.13–7.22%, respectively), with stable trends, whereas *Lactococcus* and *Enterobacter* relative abundance decreased significantly over time (relative abundance were 5.68–13.96 and 3.86–24.1%, respectively). *Lactobacillus* relative abundance was <1% during the entire ensiling period, and malic acid metabolic pathway was the most important pathway. *Enterococcus*, *Pediococcus*, and *Weissella* were negatively correlated with malic acid, with *Lactobacillus* displaying an opposite trend.

**Discussion:**

The results suggested that *Lactobacillus* activity was the lowest among lactic acid bacteria (LAB) during ensiling, which is the main reason for nettle ensiling failure, and attributable to a low capacity to compete for fermentation substrates such as malic acid against other LAB during ensiling. Additionally, anti-bacteria activity of nettle probably inhibited Enterobacter activity during ensiling. Present study probably given a solution for improve nettle silage quality through addition with malic acid.

## 1. Introduction

In recent years, the demand for feed resources has increased in the wake of livestock feed factory development, and one potential solution is exploitation of non-conventional feed sources (He et al., 2018). Nettles are distributed globally, from mild to temperate climate regions, including North Africa, parts of Asia, Europe, and North America (Kregiel et al., 2018).

Several studies have reported that nettle as feed can improve animal performance and production, for example, by enhancing rumen health in lactating cows, promoting chicken growth, improving growth and carcass quality, and enhancing cow milk quality and quantity (Humphries and Reynolds, 2014; Bhusal et al., 2022). Additionally, nettle could improve the capacity of animals to fight disease, including genital, gastrointestinal, skin, and metabolic infections (Viegi et al., 2003; Disler et al., 2014). Such benefits of nettle can be attributed to its high nutrient contents and biologically-active compounds.

Nettle leaf has high protein [30% of dry matter (DM)], fiber (10%), and fat (4%) contents, and the biologically-active compounds include terpenoids, carotenoids, vitamins, cis-9,12-linoleic, α-linolenic acids, and polyphenolic compounds (Kregiel et al., 2018). However, among the compounds above, some antibacterial compounds could inhibit microbial activity during ensiling. According to (Zhang et al., 2010), nettle ensiling is more challenging than alfalfa ensiling considering the high buffer capacity and moisture content, and low water soluble carbohydrate (WSC) concentrations in nettle; in addition, inoculation with *Lactobacillus plantarum* at a sufficient fermentation substrate level (6.68% DM of WSC) still fails to facilitate ensiling. In addition to the high moisture content in nettle, its antimicrobial activity is a potential critical factor causing its poor ensiling properties.

Several studies have observed a strong capacity of nettle to inhibit gram-positive and gram-negative bacteria that belong to *Enterococcus*, *Enterobacter*, *Bacillus*, *Lactobacillus*, etc., at the genus level, in dose dependent manners (Kregiel et al., 2018). Among the bacteria above, the *Enterococcu*s and *Lactobacillus* are the most important lactic acid bacteria (LAB) observed during ensiling (Huang et al., 2022a). Therefore, the antibacterial activity of nettle could theoretically inhibit some bacterial activity during ensiling. However, it remains unclear which compounds impair the activity of the bacteria in nettle silage, in addition to the underlying mechanisms. Therefore, in the present study, we hypothesize that nettle antimicrobial activity could inhibit some LAB activity during nettle ensiling. Additionally, the exploration of the silage metabolome could enhance our understanding of the biological processes that occur during ensiling. Therefore, the aim of the present study was to investigate the correlation between metabolites and bacterial community during nettle silage fermentation, and determine the potential factors disrupting bacterial community structure stability in nettle silage.

## 2. Materials and methods

### 2.1. Silage preparation

Nettle (*Urtica cannabina*) was harvested on 10th September 2020 from the wild in Shawan County, central mountains of Tianshan, Xinjiang, China (E 84°58′–86°24′; N 43°26′–45°20′). The nettle was left to wilt to approximately 300 g/kg fresh weight. The samples were then chopped into 2 cm stalks using a forage cutter. After manual mixing, approximately 1.0-kg samples were packed into polyethylene plastic bags equipped with a one-way air extraction valve (23 cm × 30 cm). The bags were sealed using a vacuum sealer. There were five fermentation period treatments (7, 15, 30, 60, and 90 d), with five replicates in each treatment, yielding a total of 25 bags of samples, which were stored at 24°C.

### 2.2. Characteristics of wilted and ensiled nettle

200 g silage samples (five replicates for each fermentation period treatment) were dried at 65°C for 48 h and ground to pass through a 1.0-mm sieve, and then used to determine DM content. Total nitrogen (TN) was determined using an automatic Kjeldahl nitrogen analyzer (K9840, Hanon Co. Ltd., Shandong, China), and cured protein was calculated according to the method of the Association of Official Agricultural Chemists (AOAC). WSC content was determined according to McDonald and Henderson (1964).

Fresh silage samples (20 g), with three replicates for each fermentation period treatment, were used to analyze fermentation characteristics. After using four layers of cheesecloth to filter the water-silage mixture (1:9 for v/v), the pH was measured using a portable pH meter (PHS-3C, Instrument and electrical science instrument Co. Ltd., Shanghai, China), and the supernatant was collected for use in analysis of ammonium-N (NH_4_-N) and organic acid concentrations, according to Weatherburn (1967) and Huang et al. (2022b). Briefly, for organic acid analysis, using a C18 column (150 mm × 4.6 mm, FMF-5559-EONU, FLM Scientific Instrument Co., Ltd., Guangzhou, China) for high-performance liquid chromatography (HPLC). The Na_2_HPO_4_ (1 mM, pH 2.7) and methanol were used for mobile phase A and B, respectively, with a flow rate of 0.6 ml^⋅^min^–1^, with an injection volume of 20 μL and oven temperature of 50°C. Following gradient profile: 0 min, 100% A; 5–40 min, 98% A.

### 2.3. Sequencing analysis of bacterial community

Total DNA in each sample (five replicates for each fermentation period treatment) was extracted using a commercial DNA Kit (FastDNA^®^ Spin Kit for Soil, MP Biomedicals, USA). Primers (338F: ACTCCTACGGGAGGCAGCAG; 806R: GGACTACHVGGG TWTCTAAT) targeting the V3–V4 regions of 16S rDNA were used to conduct PCR amplification. The amplicons were extracted, purified, and the raw sequences analyzed, according to (Huang et al., 2022a). The sequences were uploaded on the NCBI Sequence Read Archive (SRA) (Accession No.: PRJNA859630).

### 2.4. Metabolite analysis

The metabolites were extracted and analyzed according to (Huang et al., 2022a). Briefly, 50-g samples were taken from each fermentation treatment, with five replicates for each treatment. Metabolites were extracted using 400 μl methanol: water (4:1, v/v) solution. After precipitating the proteins, the supernatant was collected for Ultra-High Performance Liquid Chromatography-Tandem Mass Spectrometry (UHPLC-MS/MS) analysis. The UHPLC system was equipped with an ACQUITY BEH C18 column (100 mm × 2.1 mm i.d., 1.7 μm; Waters, Milford, MA, USA). The mobile phases consisted of 0.1% formic acid in water (solvent A) and 0.1% formic acid in acetonitrile: isopropanol (1:1, v/v) solution (solvent B). The sample injection volume was 2 μl and the flow rate was 0.4 ml/min. The column temperature was maintained at 40°C.

### 2.5. Statistical analysis

The nettle silage characteristics data were subjected to one-way Analysis of Variance. Data were analyzed using IBM SPSS Statistics 22 (IBM Corp., Armonk, NY, USA). Significant differences between treatments were determined using Tukey’s test at *p* < 0.05. Briefly, the bacterial community sequencing data were analyzed, and alpha diversity indices of bacterial communities were analyzed using Mothur v1.30.1^1^. Principal Coordinate Analysis (PCoA) was performed using the Vegan v2.5-3 package in R, based on Bray–Curtis dissimilarity (Schloss et al., 2009). Further analyses were carried out in the Majorbio Cloud Platform (Majorbio Bio-pharm Technology Corporation, Shanghai, China). The metabolites were analyzed by multivariate analysis, using the ropls package (v1.6.21) in R on the Majorbio Cloud Platform. Significant differences in metabolites among groups were determined based on Variable Importance Plot values > 1 and *p* < 0.05.

## 3. Results and discussion

### 3.1. Wilted and ensiled nettle characteristics

The WSC content in wilted nettle was 6.18% DM (Table 1). According to Zhang et al. (2014), the WSC content in nettle (1.7–8.7% DM) was the highest in August, and decreased with time. The samples in the present study were obtained on 10th September, similar to those of Zhang et al. (2014). To obtain well-preserved silage, the WSC content of raw material should be at least 6% (Ren et al., 2020). The pH decreased to 6.22 after 7 d of nettle ensiling, and then stabilized over time. WSC content decreased by 22.82% after 7 d of ensiling when compared with that of wilted nettle; however, WSC content stabilized in the course of ensiling from 7 to 90 d. Therefore, WSC might be a factor affecting LAB fermentation activity during nettle ensiling. Lactic acid content and pH trends were similar to that of WSC content during ensiling, and both changed after 7 d, and then stabilized afterward in the course of ensiling. Considering LAB mainly convert WSC into organic acids, pH can drop to 4.5 during ensiling (Ren et al., 2020). The results indicate that LAB fermentation could not have occurred after 7 d of ensiling.

**TABLE 1:** Ensiling characteristics of nettle (on dry matter basis %)^1^.

Item		Ensiling days					SEM	*P-*value
	**0**	**7**	**15**	**30**	**60**	**90**		
DM	31.09	29.68	29.46	29.47	29.73	30.36	0.370	0.982
CP	12.57	12.40	11.07	11.52	11.65	11.58	0.187	0.147
WSC	6.18a	4.77b	4.71b	4.96b	4.79b	4.81b	0.112	<0.001
pH	7.23a	6.22b	6.28b	6.29b	6.26b	6.34b	0.107	<0.001
LA	ND	2.28a	2.60b	2.80c	2.89c	2.97c	0.262	<0.001
AA	ND	0.65	0.62	0.62	0.74	0.74	0.086	0.166
PA	ND	0.46	0.43	0.45	0.57	0.53	0.067	0.429
AN/total *N*%	ND	5.61	5.62	5.63	5.49	5.67	0.10	0.201

^1^*n* = 3. DM, dry matter; CP, crude protein; WSC, water-soluble carbohydrates; LA, lactic acid; AA, acetic acid; PA, propionic acid; AN, ammonium-nitrogen; ND, not detected. Different lowercase letters in the same line indicate significant differences (*p* < 0.05). Butyric acid was not detected.

### 3.2. Bacterial community in ensiled nettle

**FIGURE 1:**
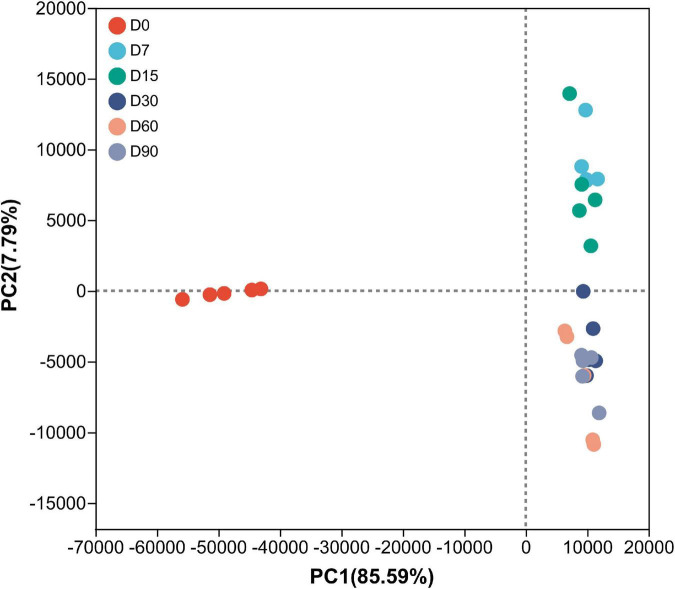
Principal-coordinate analysis (PCoA) plots based on weighted UniFrac distance for bacterial community of nettle silage. D0: wilted nettle, D7: on day 7 of fermentation of nettle silage, same as others.

As shown in Table 2, the Good’s coverage index was higher than 0.99, indicating that the degree of sequencing was sufficient for the analysis of bacterial community structure in nettle silage. Ensiling time did not have a significant effect on microbial community richness and diversity during ensiling. Similar results were observed by Guan et al. (2018), where bacterial diversity did not change significantly in the course of wheat ensiling. The results suggest that nettle silage fermentation failed, considering microbial diversity typically decreases sharply following successful fermentation (Guan et al., 2018). Additionally, as shown in Figure 1, the PCoA plots showed a clear distinction in bacterial community between 0 d fermentation and 7–60 d fermentation. According to the results, microbial community structure was stable during nettle ensiling (*R* = 0.7309, *P* = 0.001).

**TABLE 2:** Alpha diversity of bacteria community of nettle ensiling.

Days	Sobs	Shannon	Simpson	Ace	Chao	Goods coverage
0	339	1.21	0.69	381.57	385.44	0.999
7	83.6	1.71	0.24	182.03	135.8	0.999
15	92.8	1.99	0.19	162.91	136.95	0.999
30	117.2	1.99	0.22	183.06	169.54	0.999
60	116	2.14	0.21	202.99	173.71	0.999
90	110.8	1.96	0.23	174.68	160.96	0.999

**FIGURE 2:**
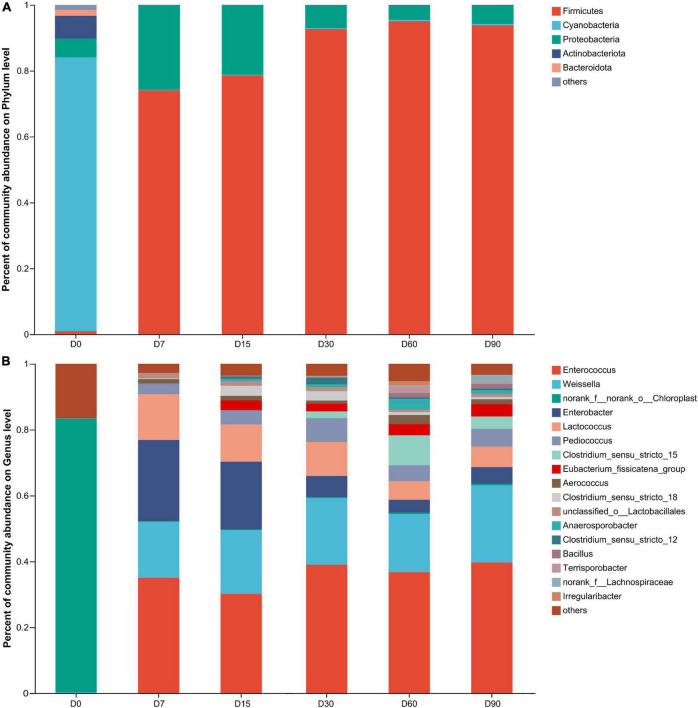
Bacterial community on phylum **(A)** and genus **(B)** level during nettle ensiling. D0: wilted nettle, D7: on day 7 of fermentation of nettle silage, same as others.

As shown in Figure 2A, at the phylum level, the bacterial community in wilted nettle was dominated by Cyanobacteria (relative abundance 83.13%), followed by Proteobacteria (5.69%) and Actinobacteria (6.90%). After ensiling, Firmicutes abundance was the highest, with a relative abundance of 74–95.06% over the entire ensiling period.

As shown in Figure 2B, at the genus level, *Enterococcus* was dominant during the entire ensiling process, with a relative abundance of 30.06–39.39%, followed by *Weissella* (17.29–23.34%), *Enterobacter* (3.86–24.1%), *Lactococcus* (5.68–13.96%), and *Pediococcus* (3.13–7.22%). In addition, the relative abundances of *Enterococcus*, *Pediococcus*, and *Weissella* stabilized during ensiling, whereas the relative abundances of *Lactococcus* and *Enterobacter* decreased in the course of ensiling (*p* < 0.05). Spherical LAB, such as *Lactococcus*, *Pediococcus*, and *Enterococcus*, exhibited greater activity during the early stages of LAB fermentation during ensiling, but were replaced by rod-shaped LAB, such as *Lactobacillus*, which have higher acid tolerance than spherical LAB (He et al., 2020). In the present study, the pH was >6.22 during nettle ensiling, which suggests that the LABs were not affected by pH during ensiling. Harvey (1965) and Boquien et al. (1988) reported that acidic environments could inactivate some LABs (*Lactobacillus lactis*, *Lactococcus*) since they decrease the activity of hexokinase, acetate kinase, and glycolytic enzyme systems, and destroy the cell membrane structure; however, such interference occurs only when the pH is <5.

Based on the method of extraction adopted for biologically active compounds, such as methanol, ethanol, butanol, and aqueous extraction, nettle exhibits varying inhibitory activity against a wide spectrum of microbial strains, at various concentrations ranging from 1 μg/ml to 72 mg/ml (Kregiel et al., 2018). A few studies have observed that animal and human diet supplemented with nettle could decrease *Lactococcu*s abundance (Asl et al., 2017; Fan et al., 2021). Furthermore, nettle extract could inhibit *Enterococcus faecalis*, and the minimal inhibitory concentration (MIC) is in the 7.5–125 μg/ml range (Kregiel et al., 2018). Unexpectedly, in the present study, *Enterococcus* activity was stable during the entire ensiling process. Therefore, during ensiling, the biologically active substances in nettle potentially inhibit only some spherical LAB, such as *Lactococcu*s, with no inhibitory effects on *Enterococcus* and *Pediococcus*. Furthermore, the contents of substances inhibiting the growth of the two LABs may be degraded due to the combined action of multiple microbes in silage.

In a previous study, *Enterobacter* activity was inhibited when pH was <4.5 during ensiling (Huang et al., 2022b). In the present study, during nettle ensiling, pH was 6.22–6.34. Theoretically, *Enterobacter* activity should not be inhibited at such pH levels. To our surprise, *Enterobacter* relative abundance was also decreased during ensiling in the present study. Similar bacterial strains obtained from different samples were inhibited differently by nettle extract obtained using the same extraction method. Ethanol nettle extract diluted with methanol exhibited strong inhibitory capacity against *Escherichia coli* from food but weak inhibitory capacity against *E. coli* from urine (Kukric et al., 2012). Therefore, the antibacterial activity of nettle could be a key factor responsible for the decreased *Enterobacter* activity during ensiling.

*Lactobacillus* becomes dominant with the prolongation of ensiling, which typically indicates good silage quality (He et al., 2020). However, in the present study, *Lactobacillus* relative abundance was <1% over the entire nettle ensiling process. Considering most spherical LAB and *Weissella* were dominant and stabilized during the entire ensiling process, the results suggest that the *Lactobacillus* abundance not adequate to confer an advantage in competition for substrate with other LAB. Additionally, in a previous study, nettle ethanol extract exhibited week inhibitory capacity against *Lactobacillus plantarum* (MIC = 72.43 mg/ml) when compared with the inhibitory capacity of some antibiotics, such as erythromycin, ampicillin, ciprofloxacin, and gentamicin (MIC = 0.078–5 μg/ml) (Kukric et al., 2012). In addition, although inoculation with *Lactobacillus* could enhance *Lactobacillus* activity during ensiling and improve silage fermentation quality (Okoye et al., 2022), such effects were not observed following inoculation of nettle silage with *Lactobacillu*s in a previous study (Zhang et al., 2010). Therefore, the results suggest that the *Lactobacillus* activity was suppressed highly during the nettle ensiling process, which could be attributed to competition for substrates with other LAB, or partly to nettle antibacterial activity.

### 3.3. Metabolites in ensiled nettle

**FIGURE 3:**
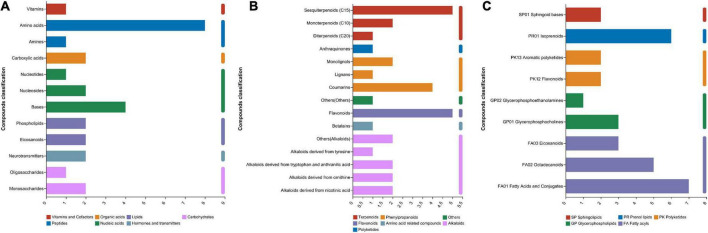
**(A)** Compounds with biological roles, **(B)** phytochemical compounds, **(C)** lipids.

**FIGURE 4:**
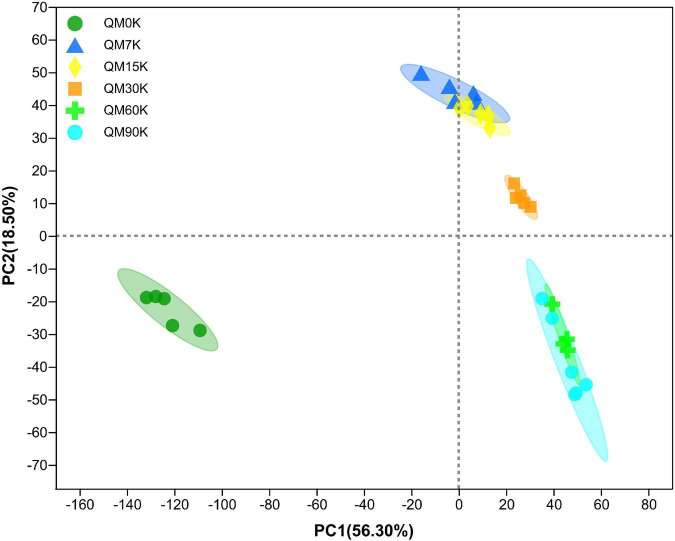
The principal component analysis score plot of the metabolites in nettle silage (QM0K: wilted nettle, QM7K: on day 7 of fermentation of nettle silage, same as others).

**FIGURE 5:**
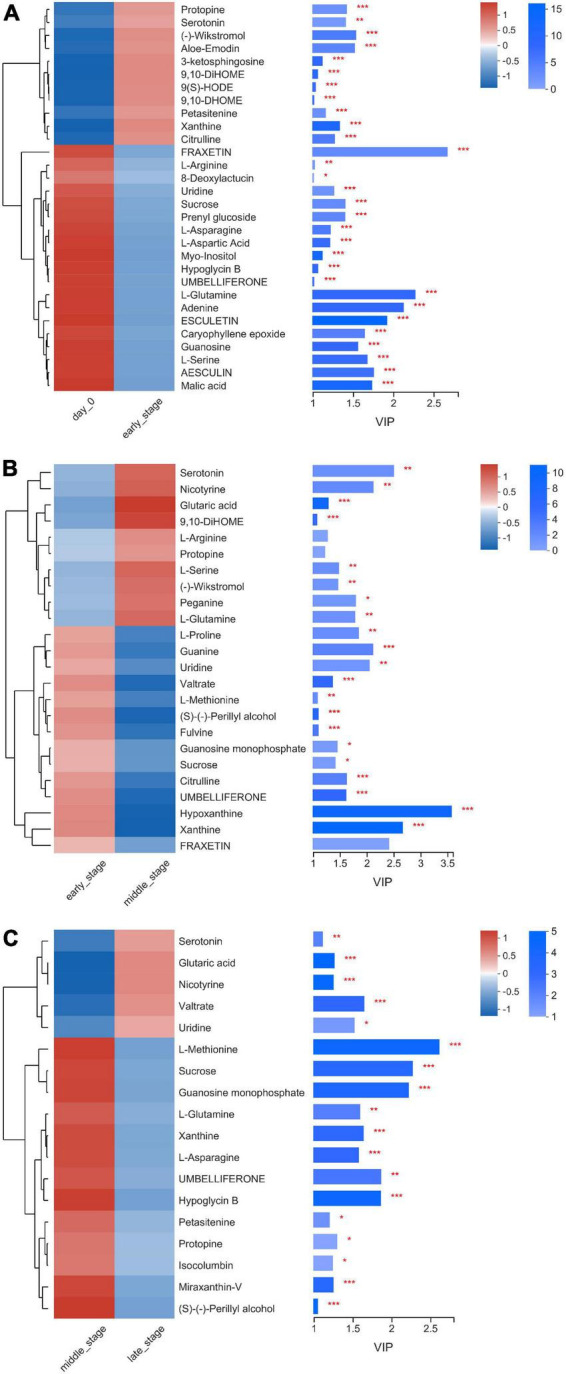
Heatmap of the differentially accumulated metabolites in nettle silage, **(A)** day 0 vs. E, **(B)** E vs. M, **(C)** M vs. L. E: early stage of fermentation (7–15d), M: middle stage of fermentation (30d), L: late stage of fermentation (60–90d).

In total, 537 metabolites were detected in the present study. Among them, 91 metabolites were annotated based on the Kyoto Encyclopedia of Genes and Genomes (KEGG) database, as shown in Figure 3. The most abundant metabolites were fatty acyls (15 metabolites), followed by peptides (9 metabolites), alkaloids (9 metabolites), terpenoids (8 metabolites), phenylpropanoids (7 metabolites), nucleic acids (7 metabolites), prenol lipids (6 metabolites), flavonoids (5 metabolites), glycerophospholipids (4 metabolites), polyketides (4 metabolites), lipids (4 metabolites), and carbohydrates (3 metabolites). There were clear distinctions in metabolites between 0 d and early stage (after 7–15 d of ensiling), early stage and middle stage (after 30 d of ensiling), and middle stage and late stage (after 60–90 d of ensiling), as shown in Figure 4. The differences in cumulative metabolites between treatment stages are shown in Figure 5.

**FIGURE 6:**
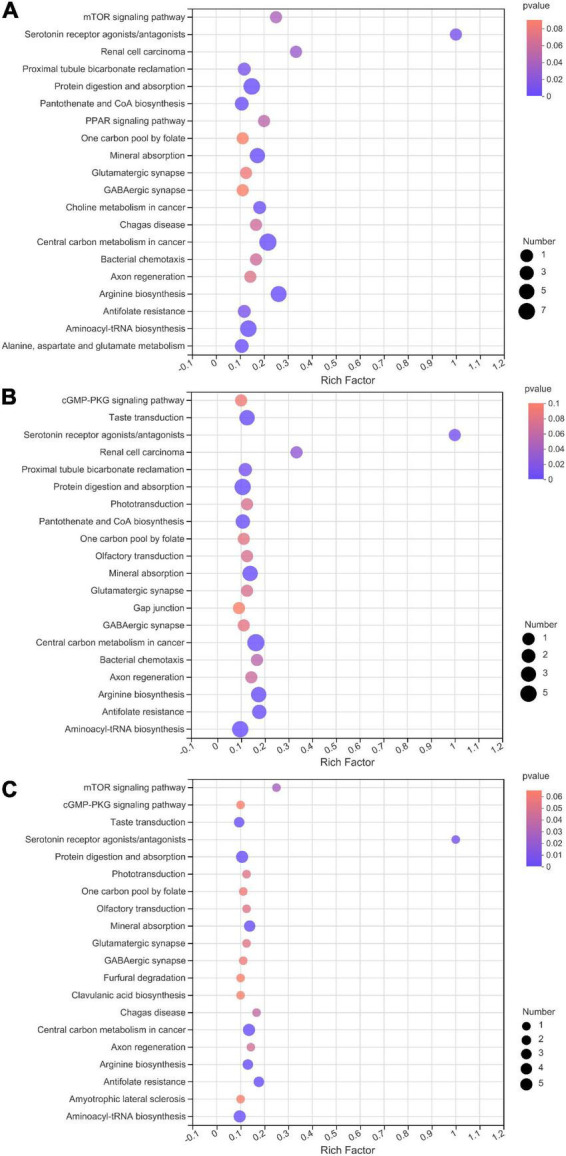
Kyoto encyclopedia of genes and genomes (KEGG) pathway enrichment analysis of differentially accumulated metabolites in nettle silage. **(A)** day 0 vs. E, **(B)** E vs. M, **(C)** M vs. L. E: early stage of fermentation (7–15d), M: middle stage of fermentation (30d), L: late stage of fermentation (60–90d).

**FIGURE 7:**
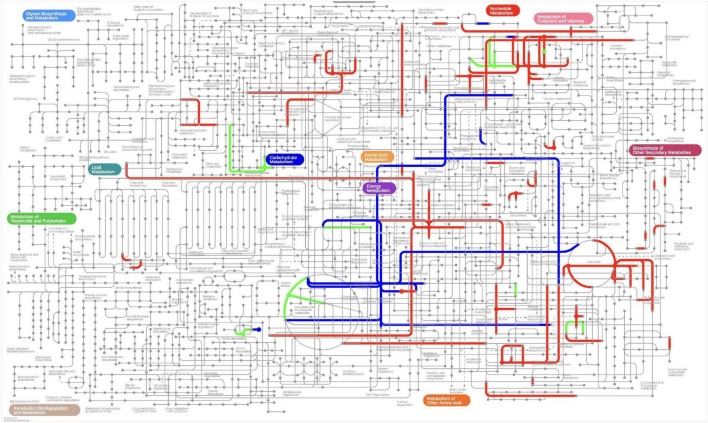
The metabolic network analysis in iPath 3.0. The different color indicates most activated metabolites and its pathways during ensiling, green indicate most activated metabolites pathway during early stage of ensiling, blue indicate most activated metabolites pathway during middle stage of ensiling, red indicate most activated metabolites pathway during entire ensiling process.

During nettle ensiling, the most active metabolic pathways were the arginine biosynthesis pathway, protein digestion and absorption pathway, aminoacyl-tRNA biosynthesis pathway, and central carbon metabolism in cancer pathway (Figure 6). In addition, there were clear distinctions in metabolites and metabolites pathways between different fermentation stages (Figure 7). During the early stage of ensiling, the citrate cycle, glycerophospholipid metabolism, and pyruvate metabolism pathways were the most active. The alanine, beta-alanine, alanine, aspartate, and glutamate metabolism pathways were relatively active during the middle stage.

Overall, the arginine biosynthesis, methane metabolism, sphingolipid metabolism, thiamine metabolism, and starch and sucrose metabolism pathways were the most active during the entire ensiling process. Among the pathways above (Figure 5), 11 metabolites were up-regulated and 19 metabolites were down- regulated during the early stage of ensiling, when compared with at day 0 of ensiling. Fatty acyls (3 metabolites) were the dominant metabolites among the 11 up-regulated metabolites and amino acids (5 metabolites) were the dominant metabolites among the 19 down-regulated metabolites. During the middle stage of ensiling, 10 metabolites were up-regulated and 14 metabolites were down-regulated when compared with the levels in the early stage of ensiling. The amino acids (3 metabolites) were dominant metabolites among the 10 up-regulated metabolites, and the amino acids (3 metabolites) and nucleic acids (3 metabolites) were dominant among the 14 down-regulated metabolites. During the late stage of ensiling, 5 metabolites were up-regulated and 13 metabolites were down-regulated, when compared with the levels in the middle stage of ensiling. The 5 up-regulated metabolites included amines (1 metabolites), organic acids (1 metabolites), lipids (1 metabolites), and nucleic acids (1 metabolites), and the amino acids (3 metabolites) were dominant among the 13 down-regulated metabolites. Among the amino acids, citrulline was up-regulated during the early stage of ensiling but down-regulated during the middle stage of ensiling; however, arginine was down-regulated during the early stage of ensiling but up-regulated during the middle stage of ensiling. The bacteria could convert arginine into ornithine through the arginine deiminase (ADI) pathway, which hydrolyzes arginine into citrulline, and then the citrulline is converted into ornithine (Cunin et al., 1987). As an end product of arginine hydrolysis, the ornithine could be released in the extracellular environment in exchange for arginine, or used as an intermediary substrate in other metabolic pathways, such as the ornithine decarboxylase pathway, to produce putrescine. Many LAB belong to the genera *Enterococcus*, *Lactobacillus*, *Leuconostoc*, and *Weissella*, and possess the ADI pathways (Benkerroum, 2016).

The present study showed that N-Acetylputrescine relative abundance did not vary between during the entire ensiling process and before ensiling (Supplementary Table 1). Putrescine is converted into spermidine through the ADI pathway (Benkerroum, 2016). Spermidine was not detected in the present study. The results suggest that arginine was converted into ornithine by bacteria mainly released in the extracellular environment during the early stage of ensiling, decreased during the middle stage of ensiling, and the conversion was terminated during the late stage of ensiling. In the present study, asparagine and glutamine were down-regulated during the early and late stages of nettle ensiling. Similar results were observed by Winters et al. (2000) during ryegrass ensiling. Moreover, the greater aerobic stability partly contributed to the preservation of the asparagine (Bai et al., 2021). In present study, methionine was down-regulated during the middle and late stages of ensiling. Asparagine and glutamine were both down-regulated during the early and late stages of ensiling. Methionine and glutamine metabolism were not affected or enhanced during ensiling, according to 16S rRNA gene-predicted functional profiles of bacterial community members (Wang et al., 2022). The results suggest that the metabolism of the amino acids was enhanced during ensiling.

In previous study, bacteria use quorum sensing (QS) to communicate with each other via autoinducer-2 (AI-2), and AI-2 biosynthesis occurs *via* the methionine catabolism cycle (Liu et al., 2018). Bacteria such as *Enterobacter* and *Lactobacillus* use AI-2 signaling mainly to respond to environmental stress and to regulate growth and metabolism (Liu et al., 2018). The results indicate that bacterial signaling activity is enhanced during the middle and late stage of ensiling. Among nucleic acids, uridine was down-regulated during the early and middle stages, but up-regulated during the late stage of ensiling. During catabolism, methionine can produce a carbon group *via* methylation, and the carbon group can be used as the source of methyl for pyrimidine, purine, and various methylated compounds (Gu et al., 2020). The results indicate that methionine metabolism is partly associated with uridine synthesis.

Four fatty acyls, including hydroxyoctadecadienoic acid (HODE), 9,10-dihydroxyoctadec-12-enoic acid (DHOME), derivative of DHOME (DiHOME), and prenyl glucoside, were involved in different metabolic pathways only during the early stage of ensiling. HODE, DHOME, and DiHOME were up-regulated whereas prenyl glucoside was down-regulated during the early stage of ensiling. HODE, DHOME, and DiHOME participate in the caryophyllene epoxide, perillyl alcohol, and valtrate biosynthesis pathways, respectively, during ensiling. Caryophyllene epoxide was down-regulated only during early stage of ensiling, perillyl alcohol was down-regulated during the middle and late stages of ensiling, whereas valtrate was down-regulated during the middle stage of ensiling but up-regulated during the late stage of ensiling. Valtrate is a small molecule anticancer agent that inhibits nuclear export signals of cells, and is extracted from plants in the family Valerianaceae (Turner et al., 2012). The increase in valtrate during the late stage of ensiling is difficult to explain.

Malic acid and gluratic acid are organic acids that participate in different metabolic pathways. In the present study, malic acid was down-regulated during the early stage of ensiling whereas glutaric acid was up-regulated during the entire ensiling process. Most LAB can convert malate into lactate through malolactic fermentation to directly produce lactate, or pyruvate, as an intermediate compound (Liu, 2003). During the early sage of nettle ensiling in the present study, *Enterococcus*, *Weissella*, *Lactococcus*, and *Pediococcus*, which belong to LAB, had a total relative abundance of 65.11–69.13%. Consequently, malic acid was fermented mainly by LAB during the early stage of ensiling. So far, no researcher has been reported glutaric acid in silage. Most studies focus on glutaric acid production *via* microbial metabolic engineering approaches, *via* four metabolic pathways. *Pseudomonas putida* is the only microbe with the natural ability to produce glutaric acid through the aminovalerate pathway *via* the degradation of L-lysine (Chae et al., 2020). In the present study, the relative abundance of *Pseudomonas* was 0.0003–0.0047%, and N2-(D-1-Carboxyethyl)-L-lysine, which is in involved in lysine degradation pathways, according to the KEGG database, was detected. The results indicate that some *Pseudomonas* strains potentially produce glutaric acid during nettle ensiling.

### 3.4. Correlation between metabolites and bacterial community structure

**FIGURE 8:**
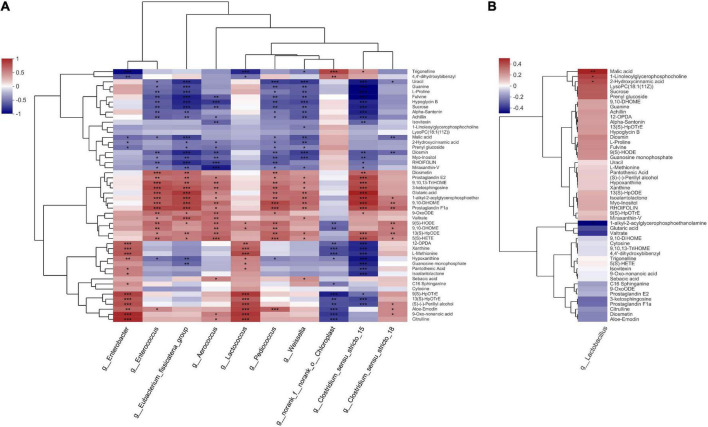
Heatmap of the correlation analysis of bacteria and metabolites in nettle silage. **(A)** Top 10 relative abundance of bacteria, **(B)** the relative abundance of *Lactobacillus.* *p < 0.05; **p < 0.01; ***p < 0.001.

The correlation between metabolites and bacterial community structure in the present study is illustrated in Figure 8. *Enterobacter* and *Lactococcus* were positively correlated with methionine (*R^2^* = 0.6285 and 0.6782, respectively, *p* < 0.05). The AI-2-dependent bio-film production *via* the methionine catabolism cycle enhances stress resistance in *E. coli* (Laganenka et al., 2016). Roe et al. (2002) observed that methionine could relieve the growth inhibition of *E. coli* caused by weak organic acid. In the present study, methionine declined steadily during the middle and late stages of ensiling, which suggests that the *Enterobacter* used methionine to tolerate stressful environmental stimuli during nettle ensiling. Similar to *Enterobacter*, *Lactococcus* could participate in methionine catabolism *via* two metabolic pathways (Bonnarme et al., 2004). In the present study, *Enterobacter* and *Lactococcus* were positively correlated with citrulline (*R^2^* = 0.7998 and 0.7309, respectively, *p* < 0.05). *Lactococcus* reportedly possesses the ADI pathway, which could produce citrulline through the hydrolysis of arginine (Benkerroum, 2016). In the present study, *Enterobacter*, *Enterococcus*, *Pediococcus*, and *Weissella* relative abundances were negatively correlated with malic acid (*R^2^* = −0.4625, −0.4082, −0.3774, and −0.5577, respectively, *p* < 0.05). Malic acid had a 23.4–37.5% inhibition rate against AI-2 activity in *E. coli*, in a dose-dependent manner, *via* penetration of the bacterial cell wall and alteration of microbial cytoplasmic pH (Almasoud et al., 2016). Considering malic acid decreased rapidly during early ensiling in the present study, and *Enterobacter* relative abundance decreased further during the middle and late ensiling stages, nettle potentially inhibited *Enterobacter* mainly *via* other compounds during ensiling. The mechanism *via* which *Lactococcus* was inhibited during nettle ensiling requires further study.

In contrast to *Enterobacter*, *Enterococcus*, and *Pediococcus* both have the capacity to catabolize malic acid. Some *Pediococcus* strains from silage, such as *Pedioccocus acidilactici* and *Pedioccocus pentosaceus* exhibit a high capacity to catabolize malic acid (98% rate of catabolism) (Fugaban et al., 2021). *Enterococcus faecalis* has two genes encoding malic enzyme, and its growth improved during glucose-malate co-metabolism (Mortera et al., 2012). In the present study, *Enterobacter*, *Pediococcus*, and *Weissella* were negatively correlated with hydroxycinnamic acid concentration (*R^2^* = −0.3749, −0.4058, and −0.5106, respectively, *p* < 0.05). During food fermentation, the competitiveness of strictly heterofermentative LAB depends on the use of external electron acceptors (Minervini et al., 2014). Filannino et al. (2014) observed that strictly heterofermentative LAB, such as *Weissella* and some *Lactobacillus* strains could metabolize hydroxycinnamic acids mainly by using hydroxycinnamic acids as external electron acceptors. However, in the present study, *Lactobacillus* was positively correlated with malic and hydroxycinnamic acid levels (*R^2^* = 0.4820 and 0.385, respectively, *p* < 0.05). Six *Lactobacillus* strains have been reported to possess *mle* and *mleS* genes, which encode malic and malolactic enzymes, respectively (Groisillier and Lonvaud-Funel, 1999). Similarly, some *Lactobacillus* strains, such as *Lactobacillus casei* and *Lactobacillus plantarum*, have been reported to have a strong capacity to convert malic acid into lactate acid through malolactic fermentation by malolactic enzyme, or decarboxylation of malic acid by malic enzyme (Olsen et al., 1991; Landete et al., 2010).

In a previous study, malic acid increased *L. plantarum* growth rate by 70%, in turn increasing biomass yield of the bacteria by 25.18%, in continuous culture in a bacterial growth medium (Passos et al., 2003). Furthermore, inoculation of silage with 1% malic increased *Lactobacillu*s relative abundance during the entire ensiling process (Tian et al., 2021). Consequently, malic acid could have a greater capacity to promote *Lactobacillus* growth during ensiling. Hydroxycinnamic acid is usually linked to hemicellulose carbohydrates of the plant cell wall through ether linkages (Novo-Uzal et al., 2011). In a previous study, four *Lactobacillus* strains with feruloyl esterase activity from ensiled corn stove exhibited a capacity to hydrolyze corn stove to release hydroxycinnamic acids (Xu et al., 2017). Theoretically, as *Pediococcus* and *Weissella* stabilized during the entire ensiling process, the hydroxycinnamic acids contents should decrease over time during ensiling due to utilization of hydroxycinnamic acids by the bacteria. However, hydroxycinnamic acid relative contents stabilized during the entire ensiling process (Supplementary Table 1), which suggests that hydroxycinnamic acid is not a critical factor in LAB succession during ensiling. Therefore, the malic acid might be the only key factor influencing LAB succession during ensiling. Considering the relative abundance of *Lactobacillus* was much less than those of other LAB, whereas *Enterococcus* and *Pediococcus* were both dominant and stabilized during the ensiling process, the results suggest that *Lactobacillus* could not compete effectively for malic acid during nettle ensiling.

## 4. Conclusion

Overall, in the present study, whole nettle plant pH after ensiling was beyond the range of high-quality silage (<4.5). During ensiling, *Enterococcus*, *Weisseria*, and *Pediococcus* relative abundances were stable, and *Enterococcus* was dominant among the bacteria. The relative abundance of *Lactobacillus* was <1%. At well-preserved silage, *Lactobacillus* usually dominant during middle and later stage of ensiling. Thus, the lowest relative abundance of *Lactobacillus* during entire ensiling process probably be the mainly reason for poor quality of nettle silage. Furthermore, malic acid, as an important LAB metabolite, decreased rapidly at the early stages of fermentation. However, the correlations between metabolites and *Lactobacillus* relative abundance had a trend opposite those of other LAB. Therefore, poor competition of *Lactobacillus* for malic acid against other LAB potentially played a key role in silage failure in the present study. The present study probably found a solution for improve nettle silage quality by addition with malic acid.

## Data availability statement

The datasets presented in this study can be found in online repositories. The names of the repository/repositories and accession number(s) can be found in this article/Supplementary material.

## Author contributions

RH: conceptualization, methodology, and writing—review and editing. FZ: resources, validation, supervision, and review and editing. YCC and YXC: resources and investigation. CM: resources and validation. SJ: resources. All authors contributed to the article and approved the submitted version.

## Footnotes

1
https://mothur.org/
